# Supplementary material for: Cell competition corrects noisy Wnt morphogen gradients to achieve robust patterning in the zebrafish embryo
Source: Nat Commun. 2019 Oct 17;10:4710. doi: 10.1038/s41467-019-12609-4 (PMC6797755; doi:10.1038/s41467-019-12609-4)
Supplement: Supplementary file 1 — Supplementary Information [file 41467_2019_12609_MOESM1_ESM.pdf]

Cell competition corrects noisy Wnt morphogen gradients to achieve robust patterning in the zebrafish embryo

Akieda *et al.*

Supplementary information:

Supplementary Figs. 1–8 and legends
Supplementary references

Supplementary Fig. 1 Related to Fig. 1

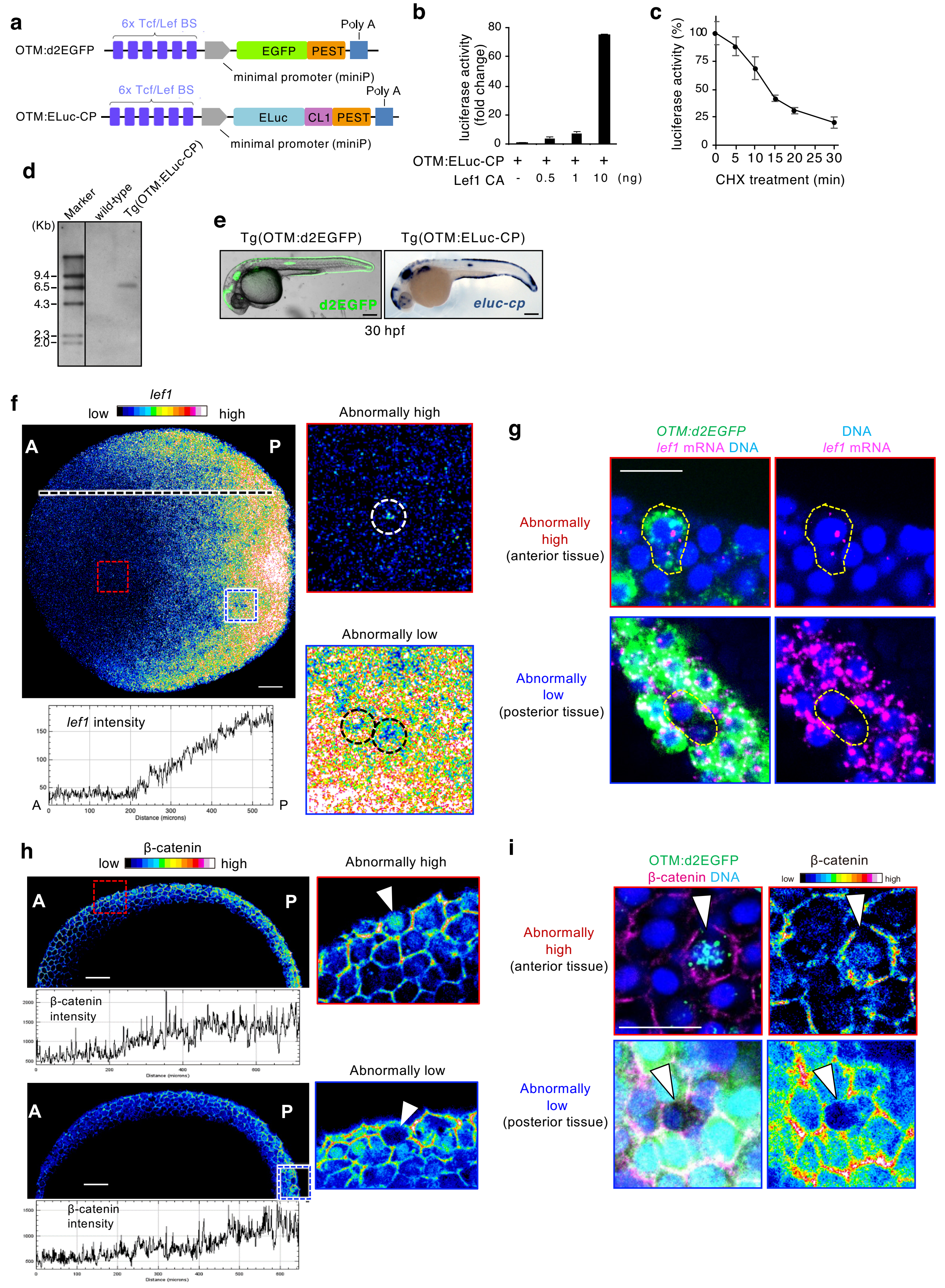

Supplementary Fig. 1 Wnt/β-catenin signalling reporter properties. Related to Fig. 1**. a** Schematic diagrams of two Wnt/β-catenin signalling-reporter constructs: OTM:d2EGFP and OTM:ELuc-CP. Tcf/Lef BS: consensus sequence of the Tcf/Lef-binding site; EGFP: enhanced green fluorescent protein; ELuc: Emerald Luciferase; PEST: degradation sequence derived from mouse ornithine decarboxylase; CL1: degradation sequence derived from yeast; Poly A: SV40 polyadenylation sequence. **b** OTM:ELuc-CP drives ELuc-CP expression in mouse neuro-2a cells in response to the activation of Wnt/β-catenin signalling. Neuro-2a cells were transfected using a reporter construct with a titrated amount of an expression plasmid-encoding Lef1 CA. Then, luciferase activity was measured. **c** The half-life of ELuc-CP is approximately 15 min. Neuro-2a cells were transfected using a reporter construct. After 36 hours, cells were treated with 100 ng/ml cycloheximide (CHX) for the indicated times. Then, luciferase activity was measured. The error bars indicate the standard deviations. **d** The OTM:ELuc-CP-transgenic zebrafish line carries a single copy of the OTM:ELuc-CP reporter. Southern blot analysis of the transgenes in the OTM:ELuc-CP transgenic zebrafish line. Genomic DNA was prepared from the tail fins of this line and used for Southern blot analysis. **e** OTM:ELuc-CP and OTM:d2EGFP are activated in same areas of zebrafish embryos. Left panel shows d2EGFP fluorescence (green) merged with bright-field of OTM:d2EGFP-transgenic zebrafish at 30 hpf (left view with the anterior side to the left). Right panel shows whole mount *in situ* hybridization of *eluc-cp* (blue) in OTM:ELuc-CP-transgenic zebrafish at 30 hpf (left view with the anterior side to the left). Scale bar, 200 μm. **f, g** Expression gradient of a Wnt/β-catenin-target gene *lef1* is noisy during zebrafish AP axis formation and *lef1* expression-noise correlates with OTM:d2EGFP reporter-noise. Confocal images showing whole-mount fluorescence in situ hybridization with *lef1* and *gfp* mRNA of wild-type (**f**) or OTM:d2EGFP (**g**) embryos. **f** Panels show fluorescence intensity and the magnified views of the noise areas. Scale bar, 20 μm. Left bottom graph shows *lef1* mRNA levels in the area indicated in left top panel dot line. **g** Naturally generated unfit Wnt/β-catenin cells (unfit cells with abnormally high or low OTM:d2EGFP activity) are enclosed with yellow dotted line circles. **h, i** Gradient of endogenous nuclear β-catenin protein levels is noisy during zebrafish AP axis formation and nuclear β-catenin-noise correlates with OTM:d2EGFP reporter-noise. Confocal images showing whole-mount immunostaining of β-catenin in wild-type (**h**) or OTM:d2EGFP (**i**) embryos. **h** Panels show fluorescence intensity and the magnified views of the noise areas. Scale bar, 20 μm. Graph shows β-catenin protein levels in the area indicated in left top panel dotted line. **i** Naturally generated unfit Wnt/β-catenin cells (unfit cells with abnormally high or low OTM:d2EGFP activity) are indicated with arrow heads.

Supplementary Fig. 2 Related to Fig. 1

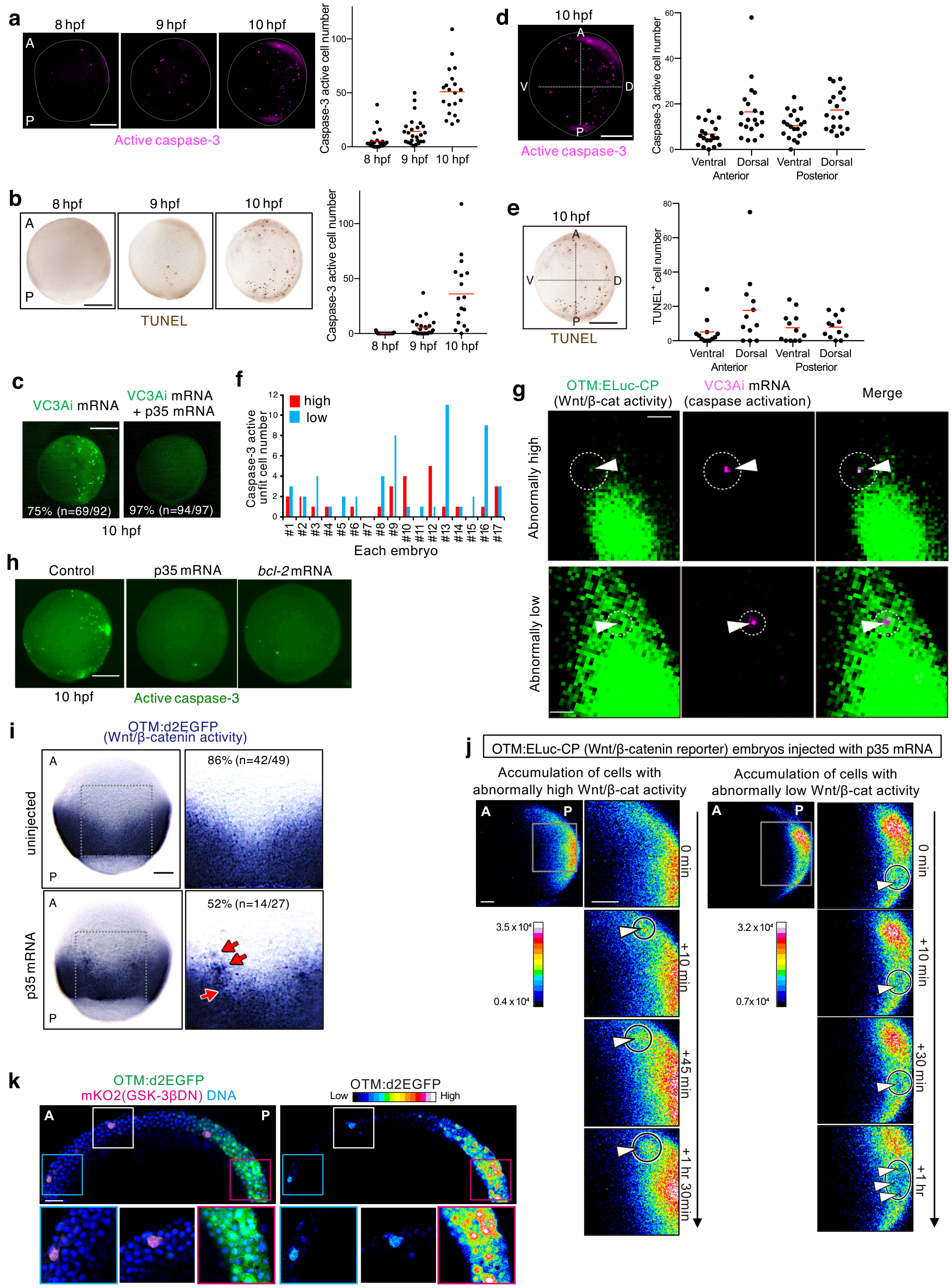

Supplementary Fig. 2 Physiological apoptosis during zebrafish AP axis formation. Related to Fig. 1**. a** Caspase-3 activation during zebrafish AP axis formation. Representative fluorescence images showing whole-mount immunostaining of active caspase-3 (magenta) in embryos maintained with normal conditions. White dotted line indicates outline of the embryo (lateral view with the anterior to the top, ventral to the left). Numbers of caspase-3-active cells in each embryo are graphed. Each dot represents one embryo (8 hpf, n = 27 embryos; 9 hpf, n = 25; 10 hpf, n = 20). Means are indicated by a horizontal bar. Scale bar, 200 µm. **b** Cell death during zebrafish AP axis formation. Same analysis as in (A) with TUNEL assay (8 hpf, n = 29 embryos; 9 hpf, n = 27; 10 hpf, n = 17). **c** Live imaging of caspase-3 activation in zebrafish embryos. Representative fluorescence images show an embryo injected with mRNA of the caspase activity fluorescent biosensor/VC3Ai[78] (250 pg) (green), or co-injected with the caspase inhibitor p35 (800 pg) (lateral with the anterior to the top, ventral to the left). Co-injection of p35 mRNA effectively reduced VC3Ai fluorescence, suggesting that the fluorescence in VC3Ai-injected embryos shows the activation of caspase. Scale bar, 200 µm. (See also Supplementary Movie 2). **d** Caspase-3 active cells appear in an irregular manner. Representative fluorescence images showing whole-mount immunostaining of active caspase-3 (magenta) in embryos maintained with normal conditions at the 10 hpf stage (lateral view with the anterior to the top, ventral to the left). A, P, V, and D indicate anterior, posterior, ventral, and dorsal, respectively. Scale bar, 200 µm. The numbers of caspase-3-active cells in each embryo are graphed. Each dot represents one embryo (n = 20 embryos). The number of caspase-3-active cells/per embryos and the position of these cells were irregular. **e** Same analysis as in (**d**) with TUNEL assay (n = 17 embryos). **f** Number of naturally generated unfit Wnt/β-catenin cells with active caspase-3 in each embryo at 8.5–9 hpf. **g** Caspase activation in naturally generated unfit cells. Representative images of OTM:ELuc-CP embryos injected with VC3Ai mRNA (magenta). OTM:ELuc-CP reporter (green) visualizes Wnt/β-catenin signalling activity. Arrows indicate naturally generated unfit Wnt/β-catenin cells with active caspase. Scale bar, 50 µm. **h** Overexpression of *bcl-2* or *p35* efficiently reduces physiologically occurring apoptosis. Representative fluorescence images showing whole-mount immunostaining of active caspase-3 (green) in embryos uninjected or injected with *bcl-2* or *p35* mRNA. Scale bar, 200 µm. **i** Apoptosis inhibition distorts the Wnt/β-catenin-gradient. Whole-mount in situ hybridization of d2EGFP in Tg(OTM:d2EGFP) embryos (dorsal view) uninjected or injected with p35 mRNA (800 ng). Magnified image of boxed area (black line; right). Embryo percentages and numbers with similar expression patterns are shown. Red arrows: ectopic activation or inactivation areas. Scale bar, 200 µm. $p<0.01$ for p35 mRNA versus control (Fisher's exact test). **j** Blocking of apoptosis induces accumulation of unfit Wnt/β-catenin cells. Time lapse images showing that unfit Wnt/β-catenin cells gradually accumulate in p35 mRNA-injected OTM:ELuc-CP embryos. Scale bars, 100 µm. Pixel area length is 6.5 µm, ≤ zebrafish deep cell diameter (~10 µm). **k** The levels of GSK3βDN-induced OTM:d2EGFP activation in the anterior tissue were similar to those of the physiological activation in the posterior tissue. Optical sagittal cross-section (dorsal side) in 8.3 hpf OTM:d2EGFP-transgenic embryos mosaically introduced with cells expressing membrane mKO2 with GSK3βDN. Left panels show fluorescence of mKO2 (magenta), OTM:d2EGFP (green), and DNA (blue). Right panels show fluorescence intensity of OTM:d2EGFP. Bottom panels show the magnified views. Scale bar, 50 µm.

Supplementary Fig. 3 Related to Fig. 2

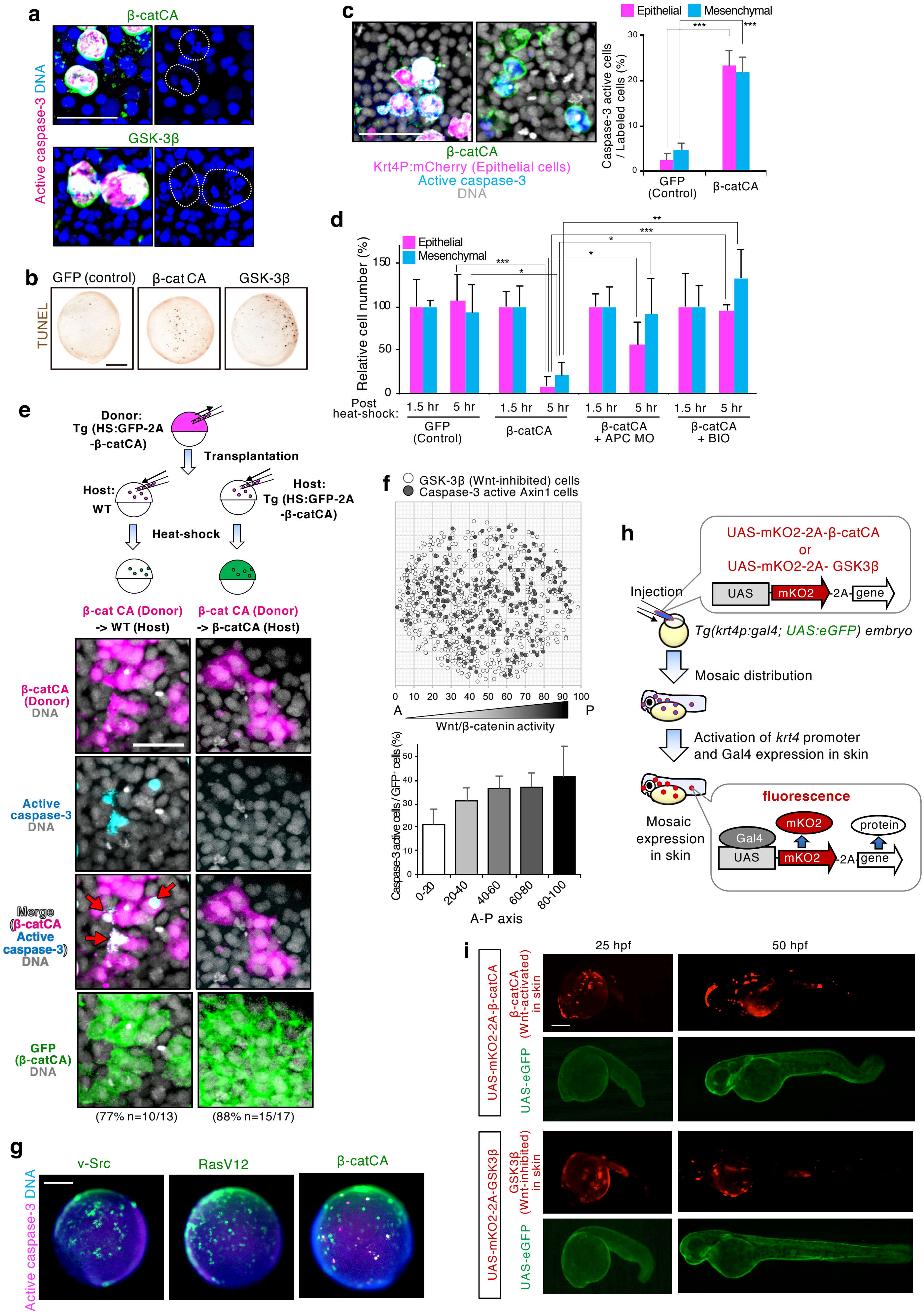

Supplementary Fig. 3 Wnt/β-catenin activity-abnormal cells require their neighbouring normal cells to undergo apoptosis. Related to Fig. 3. **a** DNA fragmentation occurs in caspase-3-active β-catCA-overexpressing cells or GSK-3β-overexpressing cells. Representative confocal fluorescence images show GFP (green), active caspase-3 (magenta), and DNA (blue) in embryos. Scale bar, 20 μm. **b** DNA fragmentation confirmed by TUNEL assay. Scale bar, 200 μm. **c** Apoptosis of β-catCA-overexpressing cells (cells with abnormally high Wnt activity) occurs both in epithelial cells ($GFP^{+}mCherry^{+}$) and mesenchymal cells ($GFP^{+}mCherry^{-}$). Embryos were co-injected with an GFP- or β-catCA (green)-expression plasmid (hsp70l:GFP or hsp70l:GFP-T2A-β-catCA) and Krt4P:mCherry plasmid, which expresses mCherry (magenta) in epithelia. $mCherry^{+}$ cells located in the epithelia, whereas $mCherry^{-}$ cells are mesenchymal cells. Confocal images showing whole-mount immunostaining of active caspase-3 (blue) in the plasmid-injected embryos. Graph shows the means ± SEM (n=8 or 9 embryos, two independent experiments) of GFP^{+} caspase-3 active cell frequencies. Scale bar, 50 μm. **d** β-catCA-overexpressing cells are eliminated from embryos, whereas upregulation of Wnt/β-catenin activity in whole embryos blocks cell elimination. Percentages of relative GFP^{+} cell numbers at 1.5 to 5 h post heat-shock in APC MO-injected or 10 μM BIO-treated embryos are shown. (n=4 or 5 embryos, two or more independent experiments) ***$p < 0.001$, **$p<0.01$ *$p < 0.05$ (*t* test). **e** Transplantation of a small number of β-catCA-expressing cells into normal embryonic tissue activates caspase-3 in the transplanted cells in contact with normal cells. Schematic illustration of the experimental design for cell transplantation. Rhodamine-dextran-labelled donor cells from Tg(hsp70l:GFP-T2A-β-catCA) embryos were transplanted into unlabelled wild-type or Tg(hsp70l:GFP-T2A-β-catCA) host embryos. Representative confocal fluorescence images show donor cells (magenta), DNA (grey), active caspase-3 (cyan), and GFP/transgene carrier (green) in transplanted embryos. The percentages of embryos showing similar phenotype and number of embryos are shown under each image. Scale bar, 50 μm. **f** Cells causing excess noise in Wnt/β-catenin-gradients efficiently undergo apoptosis. Top panels show the maps of artificially introduced cells in zebrafish embryos. Bottom graphs show the means ± SEM of caspase-3-active cell frequencies within the divided range along the AP axis (GSK-3β, n = 7 embryos, 1062 cells). **g** Signalling-abnormal cells expressing v-Src or RasV12 do not undergo apoptosis. Representative fluorescence images show whole mount immunostaining of GFP (green), active caspase-3 (magenta), and DNA (blue) in embryos mosaically introduced with cells expressing v-Src or RasV12. Scale bar, 200 μm. **h, i** Schematic illustration of experimental introduction of Wnt/β-catenin-abnormal cells in zebrafish larval skin. **h** Plasmids that express both membrane-tagged mKO2 and a Wnt/β-catenin-regulator gene in response to Gal4-mediated UAS promoter activation were injected into one-cell stage *Tg(krt4p:gal4; UAS:eGFP)* zebrafish embryos (gifts from Drs. K. Kawakami and H. Wada)[1]. Cell division during development promotes mosaic distribution of injected plasmids. *Keratin4* (*Krt4*) promoter activates Gal4 expression in larval skin (keratinocytes). Consequently, keratinocytes with the plasmid express both mKO2 and the Wnt modulator gene, resulting in the appearance of a small number of $mKO2^{+}$ Wnt/β-catenin-abnormal cells. *UAS:eGFP* is also activated in larval skin. **i** Fluorescence images show embryos artificially introduced with keratinocytes overexpressing β-catCA (Wnt/β-catenin activator) or GSK3β (Wnt/β-catenin inhibitor). Scale bar; 200 μm.

Supplementary Fig. 4. Related to Fig. 4

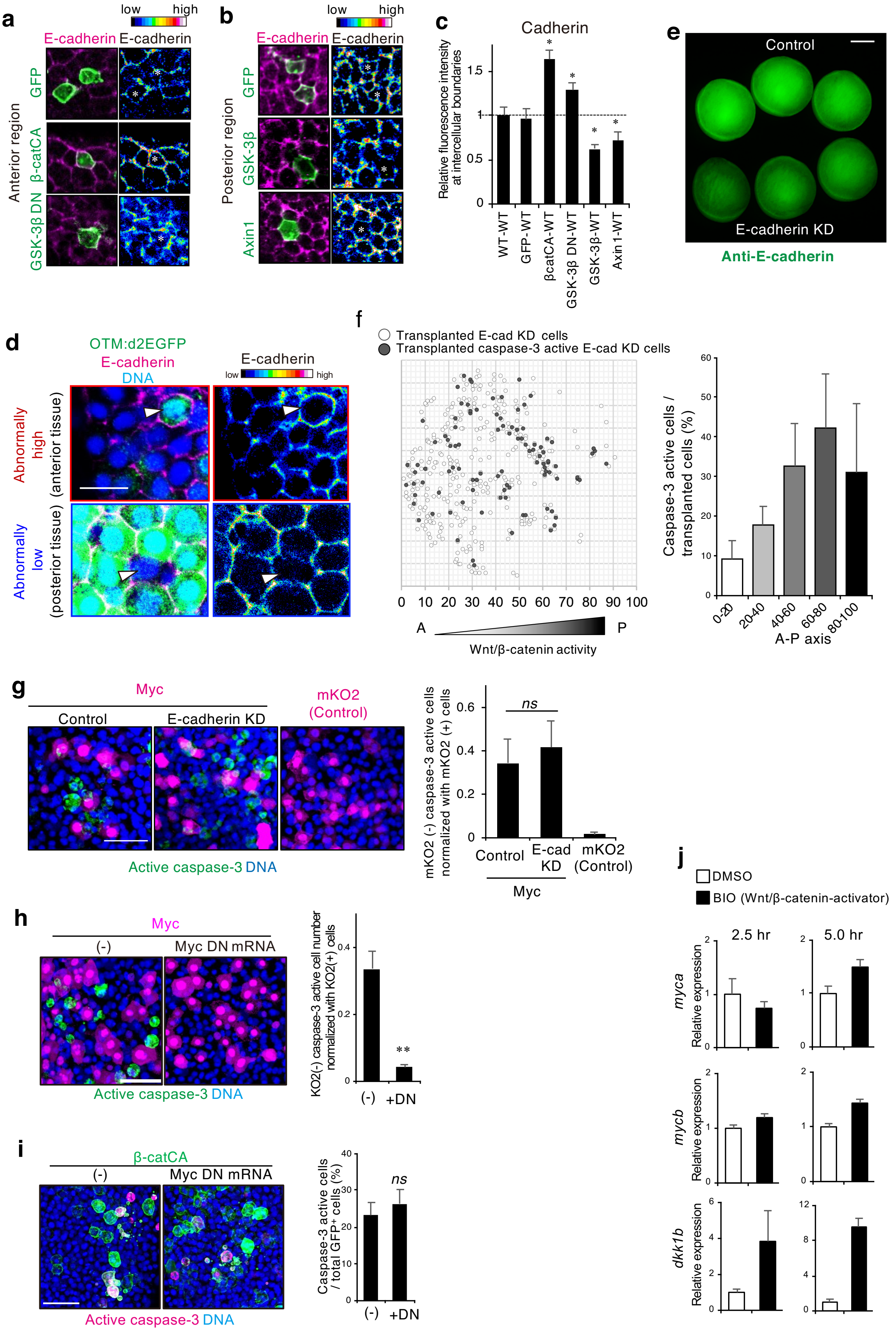

Supplementary Fig. 4 Cadherin mediates Wnt/β-catenin-unfit cell apoptosis but not Myc-driven cell competition. Related to Fig. 4. **a-c** Mosaic introduction of unfit cells with abnormal Wnt/β-catenin activity changes the levels of endogenous E-cadherin. Membrane GFP-expressing cells (GFP), Wnt/β-catenin-activated cells (cells overexpressing βcatCA or GSK3βDN with GFP), or Wnt/β-catenin-inhibited cells (cells overexpressing GSK3β or Axin1 with GFP) were mosaically introduced into zebrafish embryos. Representative confocal fluorescence images in (**a**) and (**b**) show GFP^+ cells and endogenous E-cadherin proteins (magenta and 16 colours of ImageJ) visualized by immunostaining with anti-E-cadherin. The colour bar indicates fluorescence intensity from high (red) to low (blue). Wnt/β-catenin-activation increased E-cadherin levels in the anterior region where endogenous E-cadherin levels are low, whereas Wnt/β-catenin-inhibition reduced E-cadherin levels in the posterior region where endogenous E-cadherin levels are high. **c** Fluorescence intensity (means ± SEM, n=6 or more cells) of intercellular E-cadherin staining between GFP^+ cells and neighbouring wild-type cells, normalized to the intercellular fluorescence intensity between wild-type cells in identical each image. Fluorescence intensity in two intercellular areas per cell was measured. $*p < 0.05$ (one-way ANOVA). **d** Endogenous E-cadherin protein levels change in naturally generated unfit cells with abnormally high or low Wnt/β-catenin activity. Confocal images showing whole-mount immunostaining of E-cadherin (magenta) in OTM:d2EGFP (green) embryos. Right panels show fluorescence intensity. Arrows indicate unfit cells. Scale bar, 50 μm. **e** Partial knockdown of E-cadherin (*cdh1*) in zebrafish embryos. Fluorescence image shows anti-E-cadherin immunostaining of embryos injected with 0.3 ng of control MO (upper) or E-cadherin MO (lower). Scale bar, 250 μm. E-cadherin MO reduces the fluorescence signal intensity, suggesting that this MO can reduce endogenous E-cadherin levels and that this E-cadherin antibody recognizes endogenous E-cadherin. **f** Cells causing excess noise in cadherin-gradients efficiently undergo apoptosis. Left panel shows the map of transplanted E-cadherin knockdown (E-cad KD) cells in zebrafish embryos. Right graph shows the means ± SEM of caspase-3-active cell frequencies within the divided range along the AP axis (n = 8 embryos, 474 cells). **g** High levels of Myc drives cell competition in an E-cadherin-independent manner. Representative confocal fluorescence images show mKO2 (magenta), active caspase-3 (green), and DNA (blue) in 0.3 ng of control MO (control)- or E-cadherin MO-injected (E-cad KD) embryos, which were mosaically introduced with cells overexpressing membrane mKO2 alone (mKO2) or with Myc. Scale bar, 50 μm. Means ± SEM (n=7 or 8 embryos, two independent experiments) of mKO2-negative caspase-3 active cell numbers normalized with mKO2-positive cells are shown. *ns,* $p>0.05$ (*t* test). **h, i** Dominant negative mutant of Myc (Myc-DN) blocks Myc-driven cell competition (**h**) but not Wnt/β-catenin unfit cell elimination (**i**). Representative confocal images show whole-mount immunostaining of active caspase-3 (green in **h**, magenta in **i**) in mosaic embryos expressing membrane mKO2 with Myc (magenta in **h**) or membrane GFP with β-catCA (green in **i**) and uninjected (−) or injected with Myc-DN mRNA. Scale bar, 50 μm. Graphs show the means ± SEM (n=5 or more embryos, two independent experiments) of mKO2-negative caspase-3 active cell numbers normalized with mKO2-positive cells (**h**) or of GFP-positive caspase-3 active cell numbers (**i**). $**p<0.01$; *ns*, $p>0.05$ (*t* test). **j** *Myc* genes (*myca* and *mycb*) are not immediate early genes in Wnt/β-catenin signalling in zebrafish embryos. Embryos were treated with 10 μM BIO (Wnt/ β -catenin-activator) for 2.5 or 5.0 h and then mRNA expression levels of *myca*, *mycb*, and *dkk1b* (a Wnt/β-catenin target gene) were measured by qRT-PCR.

Supplementary Fig. 5 Related to Fig. 5

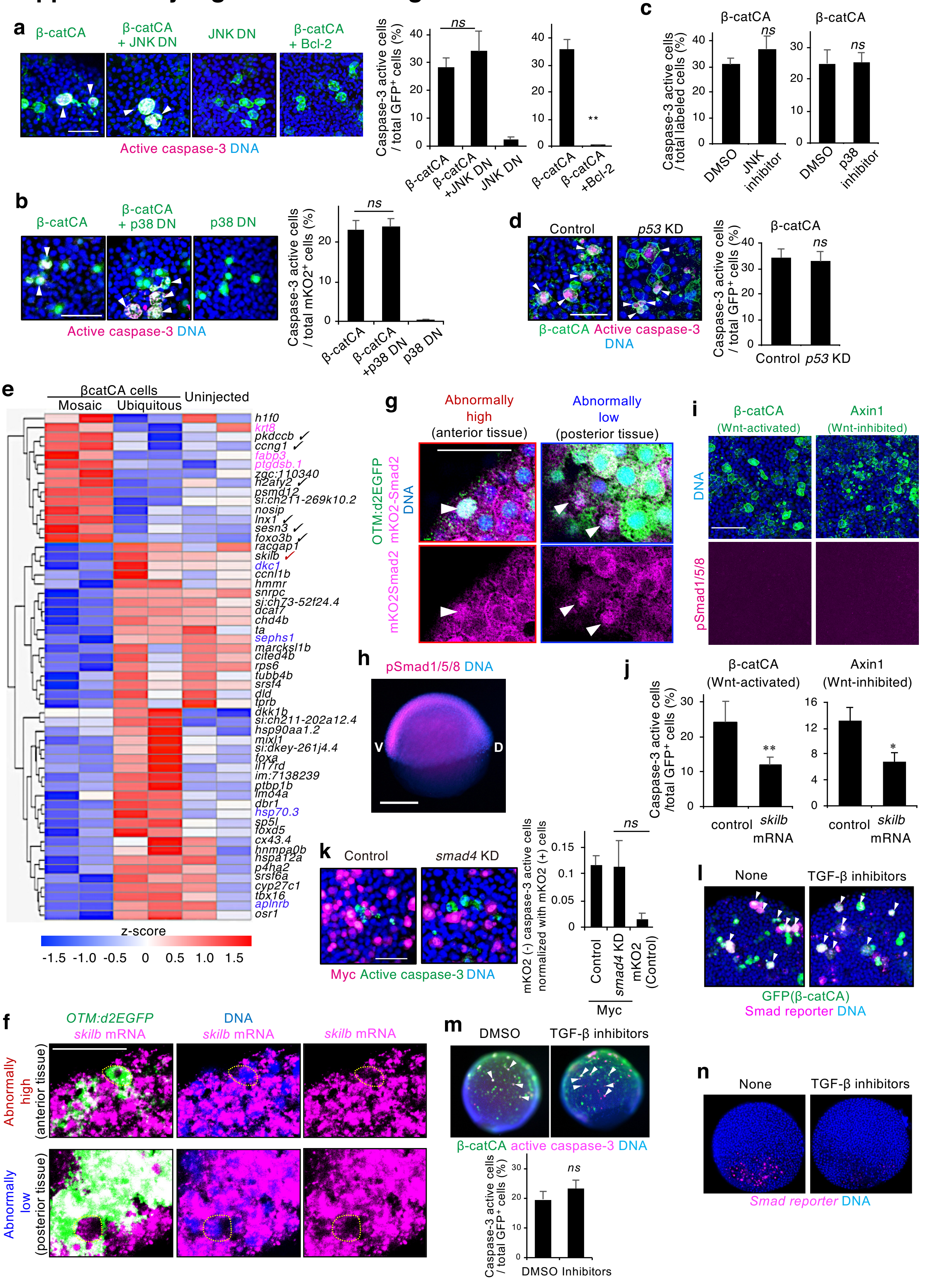

Supplementary Fig. 5 Exploration of the Wnt/β-catenin-unfit cell-killing system. Related to Fig. 5. **a** Dominant-negative mutant of JNK (JNK DN) did not block apoptosis of β-catCA-expressing cells. Representative confocal fluorescence images show GFP (green), active caspase-3 (magenta), and DNA (blue) in embryos mosaically introduced with cells expressing membrane GFP with β-catCA and JNK DN, as indicated. Means ± SEM (n=4 or more embryos, two independent experiments) of GFP^+ caspase-3 active cell frequencies are shown. Bcl-2 was used as a positive control. Injection of *bcl2* mRNA (200 pg) efficiently blocked induction of Wnt/β-catenin abnormal-cell apoptosis. **b** Dominant-negative mutant of p38 (p38 DN) did not block apoptosis of β-catCA-expressing cells. Representative confocal fluorescence images show mKO2 (green), active caspase-3 (magenta), and DNA (blue) in embryos mosaically introduced with cells expressing membrane mKO2 with β-catCA, JNK DN, and Bcl-2, as indicated. Means ± SEM (n=4 or more embryos, two independent experiments) of $mKO2^+$ caspase-3 active cell frequencies are shown. *ns*, $p>0.05$; ** $p < 0.01$ (*t* test). **c** Treatment with chemical inhibitors against JNK or p38 did not block apoptosis of β-catCA-expressing cells. Embryos were mosaically introduced with cells expressing membrane GFP with β-catCA in the presence of 0.4% DMSO, JNK inhibitor (SP600125 in DMSO, 10 μM), or p38 inhibitor (SB203580 in DMSO, 100 μM). Representative confocal fluorescence images show GFP (green), active caspase-3 (magenta), and DNA (blue) in the embryos. Means ± SEM (n=4 or more embryos, two independent experiments) of GFP^+ caspase-3 active cell frequencies are shown. Note that treatment with 10 μM SP600125 or 100 μM SB203580 induced morphological defects related to the loss of JNK or p38[2,3], respectively (Y.A. unpublished observations), suggesting that SP600125 and SB203580 blocked endogenous JNK and p38 activities, respectively. **d** Knock-down of p53 did not block apoptosis of β-catCA-expressing cells. Representative fluorescence images show GFP (green), active caspase-3 (magenta), and DNA (blue) of control MO- or *p53* MO-injected embryos mosaically introduced with cells expressing both membrane GFP and β-catCA. Means ± SEM (n=5 or more embryos, two independent experiments) of GFP^+ (β-catCA) caspase-3 active cell frequencies are shown. **e** Heat map of differentially expressed genes in Mosaic, Ubiquitous, and Uninjected embryos as evaluated by RNA-Seq (n = 2). Check marks: Smad signalling-related genes; Pink and blue genes are positive and negative ROS signalling regulators, respectively. **f, g** Endogenous *skilb* mRNA levels and Smad2 localization change in naturally generated unfit cells with abnormally high or low Wnt/β-catenin activity. Confocal images showing whole-mount fluorescent in situ hybridization of *skilb* mRNA (**f**) and immunostaining of exogenous mKO2-Smad2 (magenta) (**g**) in OTM:d2EGFP (green) embryos. Arrows indicate unfit cells. Scale bar, 50 μm. **h, i** BMP-type Smad1/5/8 are not activated in Wnt/β-catenin abnormal cells. As a positive control of immunostaining to phospho-Smad1/5/8, representative fluorescence image shows whole-mount immunostaining of anti-phospho-Smad1/5/8 (magenta) and DNA (blue) in zebrafish early embryo at 6 hpf (shield stage) (lateral view with anterior to the top, ventral to the left). V and D indicate ventral and dorsal. Scale bar, 250 μm. **i** Representative confocal fluorescence images show that GFP (green) with β-catCA or Axin1, DNA (blue) and pSmad1/5/8 (magenta). Scale bar, 50 μm. **j** Co-expression of *skilb* blocks apoptosis of β-catCA- or Axin1-expressing cells. Uninjected (control) or *skilb* mRNA (800 pg)-injected embryos were mosaically introduced with cells overexpressing membrane GFP with β-catCA or Axin1 and then caspase-3 activation was detected. Graphs show the means ± SEM (n=9 or more embryos, two independent experiments) of GFP^+ caspase-3 active cell frequencies. ** $p < 0.01$, * $p < 0.05$ (*t* test). **k** Smad4 knock-down does not block Myc-driven cell competition. Representative confocal fluorescence images show mKO2 (magenta), active caspase-3 (green), and DNA (blue) in 10 ng of control MO- or *smad4* MO-injected embryos, which were mosaically introduced with cells overexpressing membrane mKO2 with Myc. Scale bar, 200 μm. Graph shows the means ± SEM (n=8 embryos, two independent experiments) of mKO2-negative caspae-3 active cell numbers normalized with Myc-expressing cells. **l** treatment with both the TGF-β receptor inhibitor LY364947 and the Activin/Nodal receptor inhibitor SB431542 could not block Smad reporter activation in β-catCA-overexpressing cells. Representative confocal fluorescence images show GFP (green) with β-catCA, DNA (blue), and Smad reporter (SBE-luc) activity (magenta) in embryos mosaically introduced with cells

carrying SBE-luc and expressing membrane mKO2 with β-cat CA, either untreated (None) or treated with both LY364947 (100 μM) and SB431542 (200 μM). **m** Treatment with both LY364947 and SB431542 could not block apoptosis of β-catCA-overexpressing cells. Representative confocal fluorescence images show GFP (green) with β-catCA, DNA (blue), and active caspase-3 (magenta) in embryos mosaically introduced with cells expressing membrane mKO2 with β-cat CA, either untreated (None) or treated with both LY364947 (100 μM) and SB431542 (200 μM). Graphs show means ± SEM (n=6 or more embryos, two independent experiments) of GFP+ caspase-3 active cell frequencies. **n** Treatment with both LY364947 and SB431542 blocked endogenous TGF-β signalling-mediated Smad reporter activation at 4 hpf. Representative confocal fluorescence images show DNA (blue) and Smad reporter (SBE-luc) activity (magenta) in 4.3 hpf embryos introduced with SBE-luc, either untreated (None) or treated with both LY364947 (20 μM) and SB431542 (50 μM). Local activation of SBE-luc was detected in untreated embryos but not in inhibitor-treated embryos. This local activation is likely induced by endogenous TGF-β (Nodal) signalling.

Supplementary Fig. 6 Related to Fig. 6

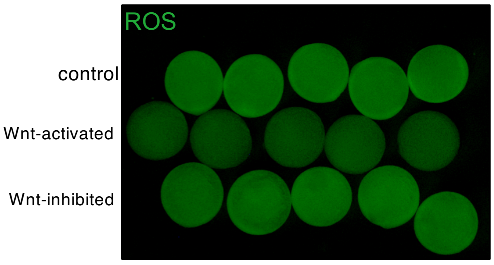

Supplementary Fig. 6 Ubiquitous Wnt/β-catenin activation or inhibition does not activate ROS production. Related to Fig. 6. Fluorescence images showing ROS probe (CellRox Green)-stained embryos untreated (top) or treated with 10 μM BIO (Wnt/β-catenin-activator) (middle) or injected with 800 ng of *dkk1b* mRNA (Wnt/β-catenin-inhibitor) (bottom). Scale bar, 200 μm. Note that ubiquitous Wnt/β-catenin activation slightly reduced endogenous ROS.

Supplementary Fig. 7 Related to Fig. 7

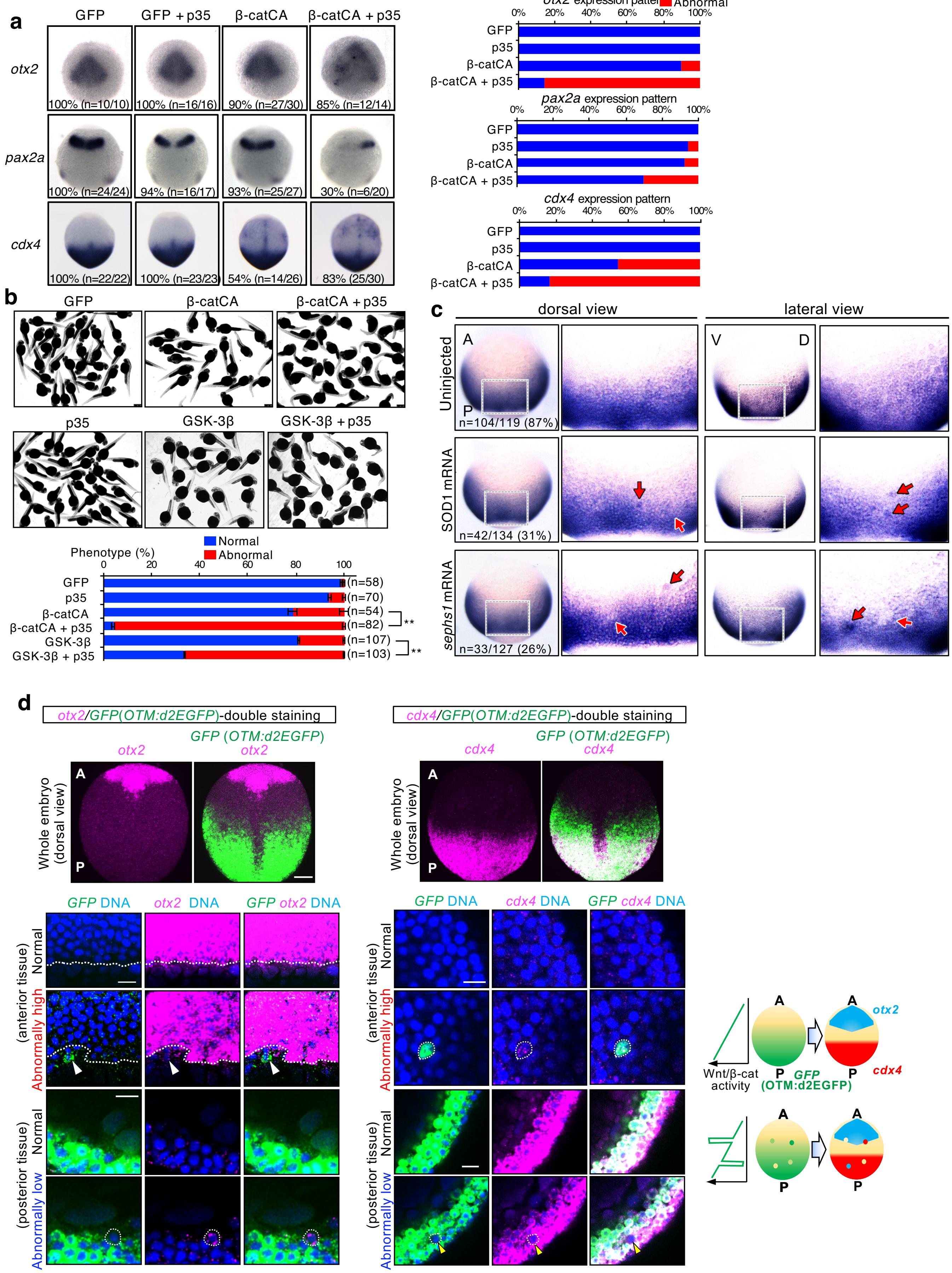

Supplementary Fig. 7 Apoptotic elimination of unfit cells is required for precise AP patterning and morphogenesis. Related to Fig. 7. **a** Inhibition of apoptosis in β-catCA-overexpressing cells disturbs AP patterning. Images show whole-mount in situ hybridization of *otx2* (marker of presumptive forebrain and midbrain), *pax2a* (marker of presumptive midbrain-hindbrain boundary), and *cdx4* (marker of presumptive spinal cord) in embryos mosaically introduced with cells expressing membrane GFP alone (GFP) or with β-catCA with or without p35. Percentages of embryos displaying abnormal expression patterns are shown. p35 co-expression in artificially introduced β-catCA-expressing cells induced ectopic expression of posterior marker *cdx4* in the anterior tissue and perturbed the expression of anterior markers *otx2* and *pax2a*. **b** Inhibition of apoptosis of β-catCA- or GSK-3β-expressing (Wnt-activated or -inhibited) cells disturbs proper morphogenesis. Embryos artificially introduced with cells overexpressing membrane GFP alone (GFP) or with β-catCA or GSK-3β with or without caspase inhibitor p35. Multi-sample images of 32 hpf larvae. Scale bar, 500 µm. Embryo percentages with normal or abnormal morphogenesis (right). Total analysed embryo numbers are shown. $^{**}p < 0.01$ (Fisher's exact test). **c** Inhibiting ROS production distorts the gradient of Wnt/β-catenin-target gene expression. Whole-mount in situ hybridization of *lef1* in zebrafish embryos (dorsal and lateral view) injected with SOD1 or *sephs1* mRNA (800 pg). Magnification of boxed area (grey line) is also shown. Embryo percentages and numbers with similar expression patterns are shown. Red arrows: ectopic activation or inactivation areas. Scale bar, 200 µm. **d** Naturally occurring Wnt-noise cells express improper pattern makers. Panels show confocal images of fluorescent whole-mount *in situ* hybridization with *GFP* (OTM:d2EGFP), *otx2* (marker of presumptive forebrain and midbrain) and *cdx4* (marker of presumptive spinal cord) in embryos. Upper panels of *in situ* hybridization represent overview of whole embryo. Scale bar, 100 µm. Lower panels represent magnified view of normal or abnormal Wnt signalling activity areas. Scale bar, 20 µm. Naturally occurring unfit cells with abnomally high or low Wnt signalling activity are indicated by arrowheads (white or yellow) or white dotted line circels. Upper schematic illustration indicates expression of *GFP* (OTM:d2EGFP) and pattern of AP tissue markers.

Supplementary Fig. 8

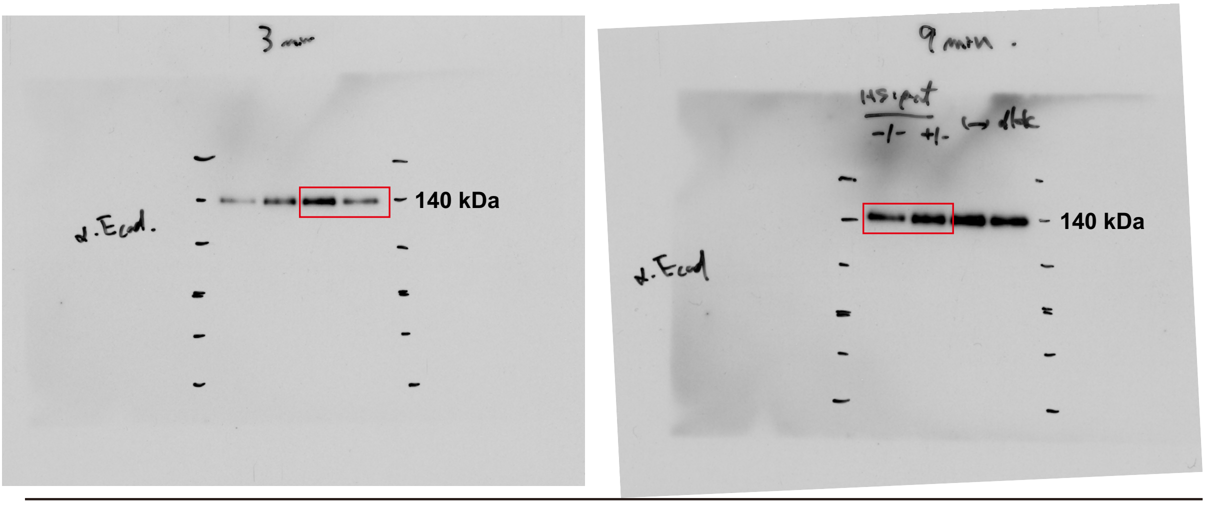

anti-E-cadherin

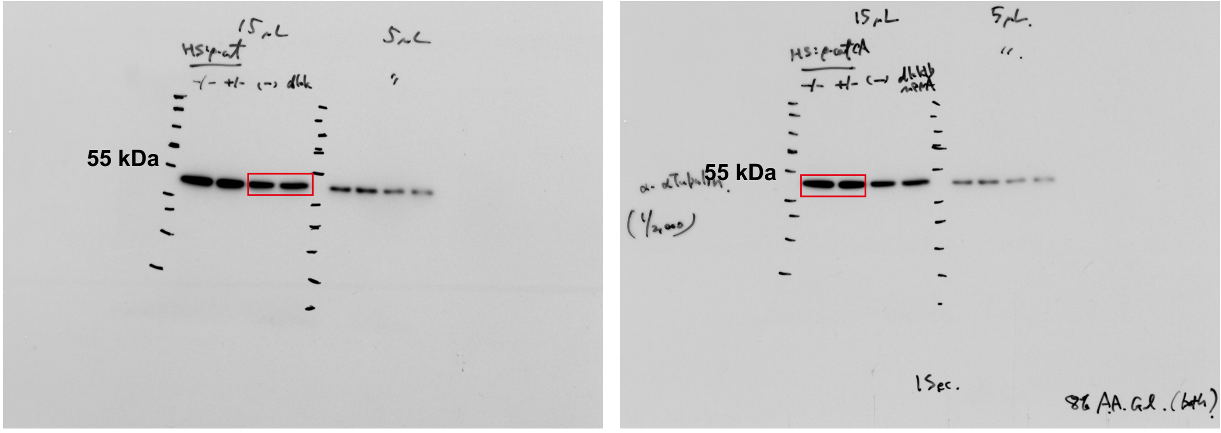

anti-α-tubulin

Supplementary Fig. 8 Uncropped scans of Western blots in Fig. 3f.

Supplementary references

1. Asakawa, K. et al. Genetic dissection of neural circuits by Tol2 transposon-mediated *Gal4* gene and enhancer trapping in zebrafish. *Proc. Natl. Acad. Sci. U. S. A.* **105**, 1255–1260 (2008).
2. Hsu, R. J., Lin, C. C., Su, Y. F. & Tsai, H. J. Dickkopf-3-related gene regulates the expression of zebrafish *myf5* gene through phosphorylated p38a-dependent Smad4 activity. *J. Biol. Chem.* **286**, 6855–6864 (2011).
3. Seo, J. et al.Negative regulation of wnt11 expression by Jnk signaling during zebrafish gastrulation. *J. Cell. Biochem.* **110**, 1022–1037 (2010).
