# Supplementary material for: Cell competition corrects noisy Wnt morphogen gradients to achieve robust patterning in the zebrafish embryo
Source: Nat Commun. 2019 Oct 17;10:4710. doi: 10.1038/s41467-019-12609-4 (PMC6797755; doi:10.1038/s41467-019-12609-4)
Supplement: Supplementary file 3 — Description of Additional Supplementary files [file 41467_2019_12609_MOESM3_ESM.docx]

Description of Additional Supplementary files

File name: Supplementary Movie 1

Description: Time-lapse imaging of Wnt/β-catenin signalling activity during zebrafish early embryogenesis. Wnt/β-catenin activity visualized by ELuc-CP luminescence (merged with bright-field image; left) in Tg(OTM:Eluc-CP) embryo (dorsal view). The luminescence intensity is shown by pseudo-colour (16 colours of ImageJ). Bar indicates luminescence intensity from high (red) to low (blue) (centre). Images were acquired every 5 min from 8 to 12 hpf. A: anterior, P: posterior. Scale bar, 200 μm. Graph shows time-lapse 3D surface plot of luminescence intensity (right).

File name: Supplementary Movie 2

Description: Live imaging of caspase-3 activation in zebrafish embryos. 3D projection of a zebrafish embryo injected with mRNA of the caspase activity fluorescent biosensor/VC3Ai (green) and membrane-tagged mKO2 (magenta) from 7 to 10 hpf. Images were acquired at 1.2 min intervals. The video is displayed at 15 frames/s. Scale bar, 100 μm.

File name: Supplementary Movie 3

Description: Behaviour of β-catCA-expressing cells in zebrafish early embryos. 3D projection of a mosaic embryos with cells overexpressing membrane GFP with β-catCA (Wnt activator) (green) around 7 hpf (left). The cell membrane was visualized with membrane mKO2 (magenta). Magnified image of boxed area (white line) (right). Images were acquired at 2-min intervals. The video is displayed at 15 frames/s. Scale bar, 100 μm.

File name: Supplementary Movie 4

Description: Behaviour of GFP-expressing control cells in zebrafish early embryos. 3D projection of a mosaic embryo with cells overexpressing membrane GFP alone (green) around 7 hpf (left). The cell membrane was visualized with membrane mKO2 (magenta). Magnified image of boxed area (white line) (right). Images were acquired at 2-min intervals. The video is displayed at 15 frames/s. Scale bar, 100 μm.
