# Supplementary material for: Cell competition corrects noisy Wnt morphogen gradients to achieve robust patterning in the zebrafish embryo
Source: Nat Commun. 2019 Oct 17;10:4710. doi: 10.1038/s41467-019-12609-4 (PMC6797755; doi:10.1038/s41467-019-12609-4)
Supplement: Supplementary file 8 — Reporting Summary [file 41467_2019_12609_MOESM8_ESM.pdf]

nature research

Corresponding author(s): Tohru Ishitani

Last updated by author(s): Sep 14, 2019

Reporting Summary

Nature Research wishes to improve the reproducibility of the work that we publish. This form provides structure for consistency and transparency in reporting. For further information on Nature Research policies, see Authors & Referees and the Editorial Policy Checklist .

Statistics

For all statistical analyses, confirm that the following items are present in the figure legend, table legend, main text, or Methods section.

n/a	Confirmed	
☐	☒	The exact sample size (*n*) for each experimental group/condition, given as a discrete number and unit of measurement
☐	☒	A statement on whether measurements were taken from distinct samples or whether the same sample was measured repeatedly
☐	☒	The statistical test(s) used AND whether they are one- or two-sided<br>*Only common tests should be described solely by name; describe more complex techniques in the Methods section.*
☒	☐	A description of all covariates tested
☒	☐	A description of any assumptions or corrections, such as tests of normality and adjustment for multiple comparisons
☐	☒	A full description of the statistical parameters including central tendency (e.g. means) or other basic estimates (e.g. regression coefficient) AND variation (e.g. standard deviation) or associated estimates of uncertainty (e.g. confidence intervals)
☐	☒	For null hypothesis testing, the test statistic (e.g. *F*, *t*, *r*) with confidence intervals, effect sizes, degrees of freedom and *P* value noted<br>*Give P values as exact values whenever suitable.*
☒	☐	For Bayesian analysis, information on the choice of priors and Markov chain Monte Carlo settings
☒	☐	For hierarchical and complex designs, identification of the appropriate level for tests and full reporting of outcomes
☒	☐	Estimates of effect sizes (e.g. Cohen's *d*, Pearson's *r*), indicating how they were calculated

Our web collection on statistics for biologists contains articles on many of the points above.

Software and code

Policy information about availability of computer code

Data collection	Stereo microscope: LAS X (Leica), Suite V3.3.0 (Leica)<br>Luminescence microscope: cellSens (Olympus)<br>Confocal laser-scanning microscope: Zen (Zeiss), FV10-ASW and FV31-S-SW (Olympus),<br>Light Sheet microscope: Zen (Zeiss)<br>qPCR: Mx3000P QPCR system (Agilent)<br>Cell sorter: FACSDiva (BD)<br>RNA-seq: HiSeq 1500 sequencing system (illumine)
Data analysis	Imaging data analysis: Fiji/ImageJ, photoshop CC (Adobe), TiLIA (Olympus), Imaris (Bitplane)<br>Statistical analysis and graphs: Excel (Microsoft), Prism7 (GraphPad)<br>RNA-seq data analysis: HTSeq, DESeq2, ChIP-X data base (http://amp.pharm.mssm.edu/lib/chea.jsp)

For manuscripts utilizing custom algorithms or software that are central to the research but not yet described in published literature, software must be made available to editors/reviewers. We strongly encourage code deposition in a community repository (e.g. GitHub). See the Nature Research guidelines for submitting code & software for further information.

Data

Policy information about availability of data

All manuscripts must include a data availability statement. This statement should provide the following information, where applicable:

- Accession codes, unique identifiers, or web links for publicly available datasets
- A list of figures that have associated raw data
- A description of any restrictions on data availability

The datasets generated during and/or analysed during the current study are available from the corresponding author on reasonable request. RNA-seq data have been deposited in the National Center for Biotechnology Information GEO database under the accession code GSE133526.

Field-specific reporting

Please select the one below that is the best fit for your research. If you are not sure, read the appropriate sections before making your selection.

☒ Life sciences ☐ Behavioural & social sciences ☐ Ecological, evolutionary & environmental sciences

For a reference copy of the document with all sections, see nature.com/documents/nr-reporting-summary-flat.pdf

Life sciences study design

All studies must disclose on these points even when the disclosure is negative.

Sample size	No statistical methods were used to predetermined sample size. Sample size was chosen by following the literature in the field.
Data exclusions	We excluded from our analyses unviable embryos. Miss injected embryos were also excluded from analysis.
Replication	All experiments were reliably reproduced.
Randomization	Embryos from zebrafish crosses were randomly allocated into experimental groups for injections and chemical inhibitor treatments.
Blinding	Since embryos from zebrafish crosses were genetically uniform and indistinguishable, blinding of the investigators was not necessary.

Reporting for specific materials, systems and methods

We require information from authors about some types of materials, experimental systems and methods used in many studies. Here, indicate whether each material, system or method listed is relevant to your study. If you are not sure if a list item applies to your research, read the appropriate section before selecting a response.

Materials & experimental systems

n/a	Involved in the study	
☐	☒	Antibodies
☐	☒	Eukaryotic cell lines
☒	☐	Palaeontology
☐	☒	Animals and other organisms
☒	☐	Human research participants
☒	☐	Clinical data

Methods

n/a	Involved in the study	
☒	☐	ChIP-seq
☐	☒	Flow cytometry
☒	☐	MRI-based neuroimaging

Antibodies

Antibodies used	Primary antibodies were as follows: anti-E-cadherin (#610181, BD Bioscience, Franklin Lakes, NJ); anti-αTubulin (#T6074, Sigma-Aldrich, St. Louis, MO); mouse anti-GFP (#A-11120, Thermo Fisher, Waltham, MA); rabbit anti-GFP (#A-11122, Thermo Fisher); anti-active caspase-3 (#559565, BD Bioscience); anti-β-catenin (#C7207 Sigma-Aldrich); anti-Sephs1 (ab96542 Abcam); and anti-mKO2 (#M168-3M, MBL, Nagoya, Japan).<br>Secondary antibodies were as follows: AlexaFluor488-conjugated anti-mouse IgG (#A-11029, Invitrogen, Waltham, MA) and anti-rabbit IgG (#A-11034, Invitrogen); AlexaFluor594-conjugated anti-mouse IgG (#A-11032, Invitrogen) and anti-rabbit IgG (#A-11037, Invitrogen); AlexaFluor647-conjugated anti-rabbit IgG (#4414, Cell Signaling Technology, Mountain View, CA)
Validation	Only commercially available antibodies were used. They were all validated by the producers.

Eukaryotic cell lines

Policy information about cell lines

Cell line source(s)	Neuro-2a cell line was gifted from Dr. Masatoshi Takeichi.
Authentication	None of the cell lines were authenticated.
Mycoplasma contamination	Cell line tested negative for mycoplasma contamination.
Commonly misidentified lines (See ICLAC register)	No commonly misidentified cell lines were used.

Animals and other organisms

Policy information about studies involving animals; ARRIVE guidelines recommended for reporting animal research

Laboratory animals	Adults zebrafish (Danio rerio) were used to obtain fertilized eggs. Zebrafish embryos and larvae used in this study were at 8 to 10 hours post fertilization and one day post fertilization, respectively.
Wild animals	We did not use wild animals.
Field-collected samples	We did not use field-collected samples.
Ethics oversight	All experimental animal care was performed in accordance with institutional and national guidelines and regulations. The study protocol was approved by the Institutional Animal Care and Use Committee of the respective universities (Kyushu University Permit# A28-005-1; Gunma University Permit# 17-051).

Note that full information on the approval of the study protocol must also be provided in the manuscript.

Flow Cytometry

Plots

Confirm that:

- [ ] The axis labels state the marker and fluorochrome used (e.g. CD4-FITC).
- [ ] The axis scales are clearly visible. Include numbers along axes only for bottom left plot of group (a 'group' is an analysis of identical markers).
- [ ] All plots are contour plots with outliers or pseudocolor plots.
- [ ] A numerical value for number of cells or percentage (with statistics) is provided.

Methodology

Sample preparation	Cell dissociation was performed as previously described (Link et al., 2006) with the following modification: 40 embryos per group at 8.3 hours post fertilization stage were placed in a solution of 2 mg/ml Pronase (Roche, Upper Bavaria, Germany) in E2 (15 mM NaCl, 0.5 mM KCl, 2.7 mM CaCl2, 1 mM MgSO4, 0.7 mM NaHCO3, 0.15 mM KH2PO4, and 0.05 mM Na2HPO4) on a 2% agar-coated dish for approximately 6 min at 28.5 oC. After dechorionation, embryos were washed with deyolking buffer (1/2 Ginzburg Fish Ringer without calcium: 55 mM NaCl, 1.8 mM KCl, and 1.25 mM NaHCO3). Embryos were transferred into a 1.5 ml tube and then the yolk was disrupted by pipetting with a 1000 µl tip. The embryos were shaken for 5 min at 1100 rpm to dissolve the yolk (Thermomixer, Eppendorf, Hamburg, Germany). Cells were pelleted at 300 g for 1 min and the supernatant discarded. Cell pellets were resuspended in FACSmax Cell Dissociation Solution (Genlantis, San Diego, CA).
Instrument	Cells were collected using FACSAriaII (BD).
Software	Samples were collected using FACSDiva software (BD).
Cell population abundance	The abundance of the relevant cell population in the post-sort fraction was not assessed.
Gating strategy	Debris was first excluded by a morphology gate based on FSC-A and SSC-A. Then, non-singlets were eliminated by a single cell gate based on FSC-H and FSC-A. GFP+ cells were gated with negative control wild-type cells.

- [ ] Tick this box to confirm that a figure exemplifying the gating strategy is provided in the Supplementary Information.
